# Impact of Cancer and Cardiovascular Disease on In-Hospital Outcomes of COVID-19 Patients: Results From the American Heart Association COVID-19 Cardiovascular Disease Registry

**DOI:** 10.21203/rs.3.rs-600795/v1

**Published:** 2021-06-11

**Authors:** David Tehrani, Xiaoyan Wang, Asim M. Rafique, Salim S Hayek, Joerg Herrmann, Tomas G. Neilan, Pooja Desai, Alicia Morgans, Juan Lopez-Mattei, Rushi V. Parikh, Eric H. Yang

**Affiliations:** UCLA: University of California Los Angeles; UCLA: University of California Los Angeles; UCLA: University of California Los Angeles; University of Michigan; Mayo Clinic College of Medicine and Science; Massachusetts General Hospital; UCLA: University of California Los Angeles; Northwestern University; MD Anderson Division of Radiation Oncology: The University of Texas MD Anderson Cancer Center Division of Radiation Oncology; UCLA: University of California Los Angeles; UCLA: University of California Los Angeles

**Keywords:** COVID-19, Cancer, malignancy, Cardiovascular diseas, mortality, severe disease, thromboembolic disease

## Abstract

**Background:**

While pre-existing cardiovascular disease (CVD) appears to be associated with poor outcomes in patients with Coronavirus Disease 2019 (COVID-19), data on patients with CVD and concomitant cancer is limited. Evaluate the effect of underlying CVD and CVD risk factors with cancer history on in-hospital mortality in those with COVID-19.

**Methods:**

Data from symptomatic adults hospitalized with COVID-19 at 86 hospitals in the US enrolled in the American Heart Association’s COVID-19 CVD Registry was analyzed. The primary exposure was cancer history. The primary outcome was in-hospital death. Multivariable logistic regression models were adjusted for demographics, CVD risk factors, and CVD. Interaction between history of cancer with concomitant CVD and CVD risk factors were tested.

**Results:**

Among 8222 patients, 892 (10.8%) had a history of cancer and 1501 (18.3%) died. Cancer history had significant interaction with CVD risk factors of age, body mass index (BMI), and smoking history, but not underlying CVD itself. History of cancer was significantly associated with increased in-hospital death (among average age and BMI patients, adjusted odds ratio [aOR]=3.60, 95% confidence interval [CI]: 2.07–6.24; p<0.0001 in those with a smoking history and aOR=1.33, 95%CI: 1.01 – 1.76; p=0.04 in non-smokers). Among the cancer subgroup, prior use of chemotherapy within 2 weeks of admission was associated with in-hospital death (aOR=1.72, 95%CI: 1.05–2.80; p=0.03). Underlying CVD demonstrated a numerical but statistically nonsignificant trend toward increased mortality (aOR=1.18, 95% CI: 0.99 – 1.41; p=0.07).

**Conclusion:**

Among hospitalized COVID-19 patients, cancer history was a predictor of in-hospital mortality. Notably, among cancer patients, recent use of chemotherapy, but not underlying CVD itself, was associated with worse survival. These findings have important implications in cancer therapy considerations and vaccine distribution in cancer patients with and without underlying CVD and CVD risk factors.

## Introduction

The novel coronavirus Severe Respiratory Syndrome Coronavirus 2 (SARS-CoV-2) has led to the ongoing and relentless global Coronavirus Disease 2019 (COVID-19) pandemic, which is now a leading cause of death in the United States (US) (1, 2). Underlying cardiovascular disease (CVD) risk factors and CVD are important prognosticators of clinical outcomes in hospitalized patients with COVID-19 (3). Patients with COVID-19 who have pre-existing malignancy and those undergoing cancer-specific therapy are disproportionately affected and represent a population at risk for worse outcomes (3–6). However, data on the impact of COVID-19 in patients with a history of prior or active cancer who also have underlying or acquired CVD from cancer-related treatments—the cardio-oncology population—is limited. Furthermore, it is important to investigate the safety of recent use of cancer therapies as observational studies early in the pandemic suggested that COVID-19 patients with cancer who are immunosuppressed as a result of their underlying active cytoreduction therapies may have increased mortality and intensive care needs compared to those without cancer (7). Although the direct impact of COVID-19 on cardio-oncology patients has not been evaluated in clinical trials, prior studies have shown that both CVD patients and cancer patients do have an increased risk for cardiac injury, which in of itself is an independent predictor of mortality in COVID-19 (8, 9). As oncologists and cardio-oncologists weigh the risk and benefits of initiating versus delaying or continuing versus stopping cancer-related therapies in light of the ongoing pandemic, it is critical to understand the impact of COVID-19 in the cancer population, and particularly among those with concomitant CVD.

To address this evidence gap, we evaluated the association of history of CVD, history of cancer, and recent cancer-related therapy with in-hospital death, as well as other adverse cardiovascular, pulmonary, and venous thromboembolic outcomes among adult patients hospitalized with COVID-19 in the multicenter American Heart Association’s (AHA) COVID-19 Cardiovascular disease (CVD) Registry powered by *Get With The Guidelines* (GWTG).

## Methods

### Data Acquisition and Source

We obtained patient-level data from 86 US hospitals in the GWTG® COVID-19 CVD registry (March through Aug 20, 2020). The GWTG® programs are provided by the AHA. The registry is a voluntary effort, and enrollment is available to all health systems in the US treating adult patients with active COVID-19. Participating hospitals are instructed to abstract detailed demographic and clinical data from the medical record of consecutive patients for the duration of the hospital stay. Data are entered into an electronic interactive case record form, which has error checks embedded within the software platform to ensure data quality and prevent erroneous data entry by trained clinical personnel. As the registry is designed as a quality improvement tool, with no intervention or participant contact, informed consent was not obtained and institutional review board exemption was obtained. Detailed methods of the registry have been previously described (10).

### Study Population, Design, and Definitions

The study cohort for the present analyses included all hospitalized adults (≥ 18 years old) with at least one documented symptom associated with confirmed active COVID-19. The primary exposure of interest was history of cancer. Patients were determined to have a history of cancer if they had active or prior solid or hematological cancer regardless of active therapy. Key variables of interest included patient’s having a history of CVD or recent chemotherapy or biologic therapy (defined as chemotherapy within 2 weeks of hospitalization). CVD was defined as a composite of history of cerebrovascular accident (CVA), history of heart failure (HF), prior myocardial infarction (MI), prior coronary artery bypass graft (CABG), or prior percutaneous coronary intervention (PCI). Other covariates included CVD risk factors [age, gender, BMI, diabetes, hypertension, hyperlipidemia, and history of smoking (cigarette use within 1 year of hospitalization)], pulmonary disease (history of chronic obstructive lung disease, interstitial lung disease, asthma, pulmonary arterial emphysema, chronic bronchitis or being treated for respiratory symptoms with inhaled or oral pharmacological therapy), and chronic kidney disease (physician diagnosis of renal insufficiency, chronic failure, or serum creatinine greater than 2.0 mg/dl).

### Study Endpoints

The primary outcome was in-hospital death. Four secondary outcomes were evaluated. Secondary outcomes included major adverse cardiovascular events (MACE—a composite of in-hospital stroke, heart failure, myocardial infarction, sustained ventricular arrhythmia, or heart block requiring temporary or permanent pacemaker), severe disease complications (composite of new need for hemodialysis, mechanical ventilation, in-hospital shock, inotropic or vasopressor use, or mechanical circulatory support use with use of intra-aortic balloon pump, percutaneous ventricular assist device, veno-arterial or veno-veno extracorporeal membrane oxygenation), or thromboembolic disease (a composite of deep vein thrombosis (DVT) or pulmonary embolism [PE]).

### Statistical Analysis

Data in tables are presented as either mean (standard deviation) or frequency (percentage) for the total study cohort and stratified by cancer history. Continuous and categorical variables were compared with the unpaired t tests or chi-square tests, respectively. Multivariable logistic regression analyses were performed for the primary and secondary outcomes; data are presented as adjusted odds ratios [aOR] with 95% confidence intervals (CI). In addition to the main effect term of history of cancer, interaction terms for history of cancer with CVD and CVD risk factors were tested for the primary outcome of in-hospital mortality. Interaction terms that were significant for in-hospital mortality were retained and presented in the final multivariable model. Similar, separate, modeling was done for each of the secondary outcomes. For the primary outcome of in-hospital mortality, a sensitivity analysis was conducted by limiting the cohort to only those with a history of cancer and generating an adjusted model with remaining covariates. All models were adjusted for body mass index (BMI), sex, race, diabetes, dyslipidemia, hypertension, chronic kidney disease, smoking history, and history of pulmonary disease. Generalized estimating equation method was used to estimate the parameters for the multivariable logistic regression models and to account for the correlated structure of the data due to clustering within hospitals. All statistical tests were two-sided, and a p-value < 0.05 was considered statistically significant. All analyses were performed with SAS version 9.4 (Cary, NC) using a deidentified data collected and coordinated via IQVIA (Parsippany, NJ) and housed on the American Heart Association Precision Medicine Platform (precision.heart.org) (10).

## Results

### Patient Population and Baseline Characteristics

Among 86 US hospitals, 8,222 patients through August 20, 2020 were included in the analysis. Among these patients, 892 (10.8%) patients had a history of cancer, 1,805 (22.0%) had a history of CVD, and 261 (3.2%) had both a history of cancer and CVD. Among cancer patients, 124 (14.0%) had recent use of chemotherapy (i.e. within 2 weeks of hospitalization). Patients with COVID-19 and a history of cancer were older and had a higher prevalence of CKD, pulmonary disease, and CVD (Table 1). Notably, compared with non-cancer patients, a similar amount of COVID-19 specific novel therapies were administered to cancer patients during their hospitalization.

### Association of Cancer with In-Hospital Mortality

**Figure 1:**
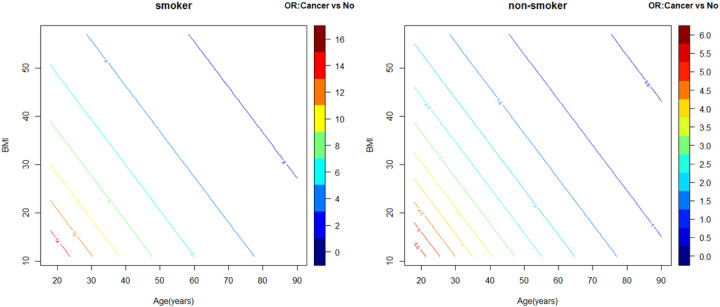
In-Hospital Mortality in Symptomatic COVID-19 Patients by Cancer Status. In multivariable analysis, age, BMI, and history of smoking were found to have significant interactions with history of cancer for the outcome of in-hospital mortality. AORs for in-hospital mortality among those with a history of cancer compared to those without a history of cancer are depicted at different values of age and BMI in smokers (Figure 1A) and non-smokers (Figure 1B). The largest effect of cancer history on mortality (darkest red aOR tier) is seen among those with a history of smoking who are younger and have lower BMIs with diminishing effects as individuals become older and more obese (blue aOR tier). aOR, adjusted Odds Ratio; BMI, Body Mass Index; CVD, Cardiovascular Disease.

In the entire cohort of adult patients hospitalized with symptomatic COVID-19, 1501 (18.3%) died during their hospitalization [median occurrence on hospital day 7 (interquartile range [IQR] 3–13 days)], 25.4% those with cancer and 17.3% of those without cancer. In univariate analysis, history of cancer was a significant predictor of in-hospital mortality (OR = 1.62, 95%CI: 1.38–1.91, p < 0.0001). Although no significant interaction between a history of cancer and a history of CVD was found, there were significant interactions between history of cancer with the CVD risk factors of age, BMI and history of smoking for in-hospital mortality. In multivariable analysis, history of cancer was a significant predictor of in-hospital mortality (among average age and BMI patients, aOR = 3.60, 95% CI: 2.07–6.24; p < 0.0001 in those with a smoking history and aOR = 1.33, 95%CI: 1.01–1.76; p = 0.04 in non-smokers). The largest effect of cancer history on in-hospital mortality was observed in those patients with a history of smoking who were younger and had lower BMIs (Fig. 1). Of note, although history of CVD was associated with in-hospital mortality in univariate analysis (OR = 2.23, 95%CI: 1.98–2.52, p = 0.010), it did not reach statistical significance for in-hospital mortality in multivariable analysis (aOR = 1.18, 95%CI: 0.99–1.41, p = 0.07). Additional significant predictors of in-hospital mortality included age, male gender, BMI among those without a history of cancer, hypertension, diabetes, chronic kidney disease, pulmonary disease, and smoking among those with a history of cancer.

Among cancer patients, in-hospital mortality occurred in 31.0% of those with a history of prior CVD as compared to 23.1% in those without a history of underlying CVD. In adjusted analysis, there was no clear association of CVD with in-hospital mortality (aOR = 1.01, 95% CI: 0.63–1.62, p = 0.95) (Table 2). In contrast, recent use of chemotherapy was significantly associated with in-hospital mortality among cancer patients (aOR = 1.72, 95%CI: 1.05–2.80, p = 0.03) (Table 2).

### Association of Cancer with Secondary Outcomes

MACE occurred in 76 (8.6%) patients with and 397 (5.5%) without a history of cancer with a median occurrence on day 1 of hospitalization (IQR 0–5 days). Although no significant interaction between history of cancer and history of CVD was found, there were significant interactions between history of cancer and gender. In multivariable analysis, history of cancer was a significant predictor of MACE among females (aOR = 1.59, 95% CI: 1.02–2.48, P = 0.04), but not males (Table 3).

Severe disease complications occurred in 1587 (21.8%) patients with and 214 (24.2%) without a history of cancer with a median occurrence on day 1 of hospitalization (IQR 0–3 days). Although no significant interaction between history of cancer and history of CVD was found for severe disease complications, there were significant interactions between history of cancer with the CVD risk factors of age and smoking history. In multivariable analysis, a history of cancer was a significant predictor of severe disease among smokers (aOR = 2.76, 95%CI: 1.98–3.86; p < 0.001) (Table 3).

Thromboembolic disease occurred in 29 (3.3%) patients with and 198 (2.7%) without a history of cancer with a median occurrence on day 6 of hospitalization (interquartile range, 1–12 days). History of cancer was not associated with thromboembolic disease in univariable or multivariable analysis (Table 3).

## Discussion

This large multicenter AHA COVID-19 CVD registry-based analysis highlights several important points regarding hospitalized adult cancer patients with symptomatic COVID-19. First, a history of cancer was found to be a significant predictor of in-hospital mortality, with its largest effect being in patients who were younger with lower BMIs and had a history of smoking. The higher rates of mortality seem to be driven by severe disease complications. Second, cancer patients who received recent chemotherapy in the prior 2 weeks to admission had increased in-hospital mortality compared with those without recent cancer-related therapy. Finally, cancer patients with concomitant pre-existing CVD had similar rates of in-hospital mortality to those cancer patients without CVD. However, CVD risk factors of age, male gender, BMI, hypertension, diabetes, and smoking were significant predictors in adjusted models.

In 2020, COVID-19, heart disease, and malignant neoplasms have been the leading causes of death in the US (2). As COVID-19 rates have exponentially increased since March 2020, the interplay between these 3 entities has become increasingly important. Small studies from China at the onset of the pandemic indicated that higher proportions of patients with a history of cancer had severe in-hospital complications and in-hospital mortality as compared to those without a history of cancer, prompting suggestions that intentional postponement of adjuvant chemotherapy or elective surgery should be considered (7, 11). With respect to cancer therapies, a small prospective cohort study reported no significant association between use of cytotoxic chemotherapy or other anticancer treatment and mortality in the setting of COVID-19 (12). A larger cohort study from the COVID-19 and Cancer Consortium similarly reported that cancer patients receiving anticancer therapies did not have higher rates of mortality, although the presence of cancer itself, whether stable/improving or progressive was independently associated with 30-day mortality (13). However, specific CVD risk factors such as hypertension and obesity, previously shown to be associated with in-hospital mortality were not adjusted for (14, 15). More recently, as the pandemic has continued to unfold, there has been more interest in evaluating risk in those with concomitant CVD and history of cancer. For example, in a retrospective study by Ganatra et al. of 2,476 patients from 4 Massachusetts-based hospitals patients with a history of both CVD (N = 414) and cancer (N = 195) had a higher short-term mortality as compared to either cancer or CVD alone (16). In addition, history of cancer was predictive of severe disease complications, while pre-existing CVD was not.

In the present study, history of cancer was a robust predictor of in-hospital mortality among hospitalized symptomatic adult COVID-19 patients. Given older age and obesity have previously been shown to be associated with mortality in the setting of COVID-19, it is not surprising that the most predominant effect of history of cancer on mortality we observed was in younger patients with lower BMIs. The increased mortality appears to be driven by increased severe disease complications in patients with history of cancer, particularly in those with a history of smoking, independent of prior pulmonary disease. Additionally, while male gender was unsurprisingly associated with higher MACE in those without cancer history, females with a history of cancer exhibited higher rates of MACE. However, interpretation is limited for MACE given the overall low event rate. Other factors that may also explain the increased mortality rate observed in cancer patients compared with non-cancer patients include: 1) residual confounding due to unaccounted for differences between cancer and non-cancer patients, such as use of vasoactive medications and code status; and 2) inadequate power to detect a difference in venous thromboembolism (VTE) given low event rates.

Venous thromboembolism (VTE) has been of particular interest in COVID-19 patients with underlying cancer given the perceived elevated risk of VTE from both the hypercoagulable/inflammatory milieu of COVID-19 and cancer itself. Data has shown that elevated D-dimers levels are independently associated with thrombotic events, and subsequently those with thrombotic events have higher all-cause mortality (17). However, the prevalence of VTE in COVID-19 cancer patients is not clear. Ganatra et al reported VTE in 10% of cancer patients admitted with COVID-19, whereas we observed VTE in only 3% of cancer patients with COVID-19 (16). This may be an underestimation given other smaller studies suggest higher rates even in those without a history of cancer (18), and may be a refection of low rates of systematic surveillance and suspicion without confirmation in critically ill patients.

To the best of our knowledge, this AHA COVID-19 registry-based study is the largest multicenter prospective analysis to date to evaluate the effect of recent use of cancer-related therapy in hospitalized symptomatic COVID-19 patients. In contrast to prior studies, among cancer patients, we observed a strong independent association of cancer-specific therapy with in-hospital mortality. Importantly, cancer patients with underlying CVD did not have higher in-hospital mortality rates compared with those without CVD. Taken together, these findings suggest that oncologists and cardio-oncologists alike should discuss with their patients the optimal timing for and possible delay of cancer-related therapy in areas where COVID-19 viral transmission is high and hospital resources are limited, but that the presence of CVD should not be a major determinant of therapy in the cardio-oncology population. Multiple societal documents have similarly discussed the need to potentially curtailing the frequency of cardiotoxicity surveillance for selected patients (19–21).

This study has important limitations. First, the AHA CVD COVID-19 registry does not provide granular data regarding the type of cancer, status of cancer (relapse, remission, etc.), or cancer-specific therapies that patients are receiving to allow for stratification of cancer severity. Second, the initial registry data did not include information regarding code status and advanced directives, which may be disproportionately high in those with cancer and thus possibly impact in-hospital outcomes. Third, these data only pertain to in-hospital outcomes and thus post-discharge outcomes are not known. Lastly, the voluntary hospital participation in the registry is weighted toward urban academically affiliated institutions, and so the results may not be generalizable; however, we adjusted for site-specific clustering of outcomes to address this issue.

## Conclusion

In summary, in the large national AHA COVID-19 CVD registry, history of cancer is a significant predictor of in-hospital mortality. Notably, among cancer patients, those with underlying CVD had similar outcomes to those without CVD, while recent use of cancer-related therapy was significantly associated with in hospital mortality. Further studies focusing on malignancy type and specific treatments, and their interactions with concomitant CVD and CVD risk factors, are warranted to identify vulnerable subgroups of the cancer population that may benefit from aggressive interventions during the COVID-19 pandemic including earlier prioritization for vaccine initiation.

## Figures and Tables

**Table 1:** Baseline Characteristics of Symptomatic Adult Patients Hospitalized with COVID-19

		History of Cancer		
Characteristic		No (N = 7330)	Yes (N = 892)	p
Age (years)		60.4 (17.4)	70.6 (14.1)	< 0.001
Female Sex		3231 (44.1)	421 (47.2)	0.077
BMI (kg/m^2^)		30.6 (8.2)	28.5 (7.7)	< 0.001
Race	White	4522 (61.7)	598 (67.0)	0.009
	Black	1736 (23.7)	194 (21.8)	
	Asian/Pacific Islander	469 (6.4)	46 (5.2)	
	Other	603 (8.2)	54 (6.1)	
Payment Source	Uninsured	521 (7.1)	22 (2.5)	< 0.001
	Private	3946 (54.0)	459 (51.6)	
	Medicare	1267 (17.4)	274 (30.7)	
	Medicaid	1310 (17.9)	124 (13.9)	
	Other	259 (3.6)	11 (1.2)	
Atrial Fibrillation/Flutter		626 (8.5)	131 (14.7)	< 0.001
Diabetes Mellitus		2722 (37.1)	328 (36.8)	0.832
Pulmonary Disease		2522 (34.4)	380 (42.6)	< 0.001
Chronic Kidney Disease		928 (12.7)	146 (16.4)	0.002
Hypertension		4287 (58.5)	616 (69.1)	< 0.001
Smoking History		461 (6.3)	61 (6.8)	0.53
Pulmonary Disease		1305 (17.8)	203 (22.8)	<0.001
Admission Symptoms				
	Fever/Chills	4823 (65.8)	550 (61.7)	0.014
	Cough	4867 (66.4)	534 (59.9)	< 0.001
	Shortness of Breath	4552 (62.1)	513 (57.5)	0.008
	Fatigue	1845 (25.2)	232 (26.0)	0.59
	Headache	722 (9.9)	49 (5.49)	< 0.001
	Myalgia	1580 (21.6)	188 (21.1)	0.74
	Sore Throat	506 (6.9)	53 (5.94)	0.28
	Nasal Congestion	375 (5.1)	53 (5.94)	0.30
	Nausea/Vomiting/Diarrhea	2066 (28.2)	254 (28.5)	0.86
	Loss of Smell/Taste	303 (4.1)	44 (4.9)	0.26
	Confusion or AMS	770 (10.5)	145 (16.3)	< 0.001
COVID-19 Therapies				
	Corticosteroid	1547 (21.8)	203 (24.7)	0.056
	Immunoglobulin	53 (0.7)	12 (1.4)	0.05
	Convalescent serum	195 (2.7)	27 (3.1)	0.54
	Ritonavir/Lopinavir	76 (1.1)	7 (0.9)	0.60
	Remdesivir	565 (7.8)	105 (11.9)	< 0.001
	Tocilizumab	523 (7.2)	62 (7.0)	0.82
Chemotherapy/biologics		14 (0.19)	124 (14.0)	< 0.001
History of CVD		1544 (21.1)	261 (29.3)	< 0.001

Values are depicted as mean (standard deviation) or percentage (N) when appropriate.

BMI, Body Mass Index; CVD, Cardiovascular Disease; AMS, Altered Mental Status

**Table 2:** Predictors of in-hospital all-cause mortality in Cancer Patients Admitted with Symptomatic COVID-19

		Multivariable Analysis	
	OR	95% CI	p
Age (per 10 years)	1.43	1.22–1.67	< 0.0001
Female Gender	0.59	0.44–0.79	0.001
BMI (per 10 units)	0.99	0.78–1.25	0.92
Black Race*	1.15	0.80–1.65	0.46
Asian or Pacific Islander Race*	1.43	0.80–2.53	0.22
Undetermined or Other Race*	1.05	0.66–1.70	0.83
Diabetes	1.13	0.82–1.57	0.45
Hypertension	1.34	0.88–2.04	0.17
Hyperlipidemia	1.12	0.81–1.53	0.50
Smoking History	2.63	1.54–4.48	< 0.0001
CKD	0.85	0.60–1.20	0.35
Pulmonary Disease	1.47	1.03–2.09	0.033
CVD History	1.01	0.63–1.62	0.95
Chemotherapy or Biologic Therapy	1.72	1.05–2.80	0.030

* As compared to Caucasian white race

BMI, Body Mass Index; CVD, Cardiovascular Disease; CI, Confidence Interval; CKD, Chronic Kidney Disease; OR, Odds Ratio.

**Table 3:** Cardiovascular Disease and Cancer History Association with Major Complications in Patients with Symptomatic COVID-19 Admissions

Outcome of Interest			Univariable Analysis			Multivariable Analysis	
		OR	95% CI	p	OR	95% CI	p
MACE							
	CVD History	2.06	1.69–2.50	<0.001	1.32	1.05–1.67	0.018
	Cancer History*	1.63	1.26–2.11	<0.001			
	among Males				0.85	0.65–1.12	0.26
	among Females				1.59	1.02–2.48	0.039
Severe Disease Complication							
	CVD History	1.26	1.12–1.41	<0.0001	0.91	0.78–1.07	0.26
	Cancer History^†^	1.15	0.99–1.34	0.062			
	among Smokers^‡^				2.76	1.98–3.86	<0.001
	among Non-Smoker^‡^				1.12	0.91–1.37	0.29
Thromboembolic Event							
	CVD History	1.01	0.73–1.38	0.97	0.93	0.68–1.28	0.653
	Cancer History	1.21	0.82–1.80	0.34	0.86	0.55–1.32	0.482

All multivariable models were adjusted for age, gender, body mass index, race, diabetes, hypertension, hyperlipidemia, smoking history, chronic kidney disease, and pulmonary disease.

* The following interaction term is found to be significant in the multivariable logistic regression analysis for the outcome of MACE and retained in the final model: Gender*Cancer History (p = 0.03).

† The following interaction terms are found to be significant in the multivariable logistic regression analysis for the outcome of severe disease complications and retained in the final model: Age*Cancer History (p = 0.01) and Smoking History*Cancer History (p = 0.003).

‡ Evaluated at an average age (61.5 years) in the study cohort CVD, Cardiovascular Disease
